# A receptor-based assay to study the sweet and bitter tastes of sweeteners and binary sweet blends: the SWEET project

**DOI:** 10.1093/chemse/bjae041

**Published:** 2024-11-08

**Authors:** Christine Belloir, Mathilde Jeannin, Adeline Karolkowski, Corey Scott, Loïc Briand

**Affiliations:** Centre des Sciences du Goût et de l’Alimentation, CNRS, INRAE, Institut Agro, Université de Bourgogne, F-21000 Dijon, France; Centre des Sciences du Goût et de l’Alimentation, CNRS, INRAE, Institut Agro, Université de Bourgogne, F-21000 Dijon, France; Centre des Sciences du Goût et de l’Alimentation, CNRS, INRAE, Institut Agro, Université de Bourgogne, F-21000 Dijon, France; Cargill Core Research and Development, Plymouth, MN, United States; Centre des Sciences du Goût et de l’Alimentation, CNRS, INRAE, Institut Agro, Université de Bourgogne, F-21000 Dijon, France

**Keywords:** bitterness, blend, sweeteners, sweetness, synergy, taste receptor

## Abstract

Sweeteners are used in the food industry to provide sweetness similar to sugar and to decrease the caloric intake and risks associated with obesity. However, some sweeteners are characterized by bitter, metallic and other off-tastes. Sensory and cellular studies have demonstrated synergies between sweetener blends, which are responsible for enhancing sweetness. This study aimed to identify new sweetener blends that are able to enhance sweetness intensity without causing bitter off-taste using in vitro functional expression of taste receptors. The dose–response of the sweet taste receptor (TAS1R2/TAS1R3) was determined for sucrose and 9 sweeteners and was consistent with their sweetness potency. Stimulation of TAS1R2/TAS1R3 by 6 binary sweetener blends confirmed 3 known synergies determined by sensory analysis, including sucralose/acesulfame-K, rebaudioside A/erythritol and rebaudioside A/thaumatin, and revealed 2 new synergies, known as, neotame/D-allulose and mogroside V/thaumatin. No synergy was observed for the rebaudioside M/mogroside V blend, probably due to their common binding sites on the sweet taste receptor. The ability of the 9 selected sweeteners to activate the 25 human bitter taste receptors (TAS2Rs) was tested. The cellular-based assay demonstrated that sucralose, acesulfame-K, rebaudioside A, mogroside V and D-allulose activated at least 2 TAS2Rs. Sucralose, acesulfame-K and rebaudioside A exhibited lower EC_50_ values for TAS1R2/TAS1R3 than for TAS2Rs, which may explain their absence of bitter off-taste at low concentrations, unlike mogroside V and D-allulose. Our data provide a receptor-based understanding of the complex synergies among sweetener blends and an effective approach for testing new sweeteners while avoiding the activation of TAS2Rs.

## 1. Introduction

The combination of genetic, hormonal, environmental, and behavioral (for example, the increase of daily sugar consumption) factors is mainly responsible for the rising incidence of obesity in many developed countries, which correspondingly increases the risk of noncommunicable diseases, including type II diabetes and cardiovascular diseases (World Health Organization 2003). A frequently recommended approach for reducing sugar consumption is to partially or totally replace sugar with low or noncaloric sweeteners. Many food and beverage companies use a plethora of sweeteners such as saccharin, aspartame, cyclamate, sucralose, and acesulfame-K (Ace-K) (O’Brien-Nabors 2001; DuBois and Prakash 2012; Belloir et al. 2017).

Sweet-tasting compounds (such as sugars and natural and synthetic sweeteners) are detected by a single heterodimeric receptor composed of 2 class C G-protein-coupled receptors (GPCRs), known as TAS1R2 and TAS1R3, which are expressed on oral taste buds (Nelson et al. 2001; Li et al. 2002). Each subunit of the receptor is composed of 3 domains: a large N-terminal extracellular region composed of a Venus flytrap domain (VFT) and a cysteine-rich domain (CRD) linked to the 7-helix transmembrane domain (7TM). TAS1R2-VFT is the major ligand-binding site for most sweet-tasting compounds, including natural sugars (glucose, fructose, and sucrose) and high-potency sweeteners such as sucralose, aspartame, neotame, Ace-K, saccharin, and steviosides (Nie et al. 2005; Zhang et al. 2010; Masuda et al. 2012; Belloir et al. 2017). Although the 3D structure of the human sweet taste receptor is not known, the structure of the VFT of the medaka fish sweet taste receptor has been determined via crystallographic analysis (Nuemket et al. 2017). In addition, a limited number of sweet-tasting compounds, such as perillartine and S-819, which are semisynthetic sweeteners, interact with the 7TM domain of TAS1R2 (Servant et al. 2011; Cai et al. 2016; Zhao et al. 2018). The VTF domain of TAS1R3 subunit binds to natural sugars (such as glucose and sucrose) and chlorodeoxysugar sucralose (Nie et al. 2005; Maîtrepierre et al. 2012). Cyclamate and neohesperidin dihydrochalcone (NHDC) are known to interact with the TAS1R3-7TM domain (Jiang et al. 2005; Winnig et al. 2007), whereas TAS1R3-CRD is involved in the receptor response of the 2 sweet-tasting proteins, brazzein and thaumatin (Jiang et al. 2004). The presence of multiple binding sites in the sweet taste receptor TAS1R2/TAS1R3 explains the chemical diversity of sweet-tasting compounds and the sweet taste synergy that occurs with blends of sweeteners involving allosteric effects (Cui et al. 2006; Servant et al. 2011; Wang et al. 2022). For example, NHDC and cyclamate synergistically enhance the response of the human sweet taste receptor to sucrose (Fujiwara et al. 2012). Sucrose interacts with the VTF domains of TAS1R2 and TAS1R3, whereas NHDC and cyclamate bind TAS1R3-7TM. Furthermore, sensory analysis has demonstrated synergism among binary mixtures of sweeteners (Vincent et al. 1955; Frank et al. 1989; Ayya and Lawless 1992; Schiffman et al. 1995a; Choi and Chung 2015; Reyes et al. 2019). Thus, the combination of Ace-K and alitame, aspartame, cyclamate, stevioside, or sucralose has synergistic effects (Schiffman et al. 1995a), whereas blends of Ace-K and saccharin or thaumatin have suppressive effects (Reyes et al. 2019).

Despite the interest in sweeteners, they are also responsible for off-flavors, particularly bitterness (Schiffman et al. 1995b; Kamerud and Delwiche 2007; Roudnitzky et al. 2011; Fujimaru et al. 2012; Allen, McGeary, and Hayes 2013; Allen, McGeary, Knopik, et al. 2013), which limits their use. The bitterness of these compounds is due to their ability to activate a family of 25 type 2 taste receptors (TAS2Rs). TAS2Rs are GPCRs that exhibit various molecular receptive ranges (Meyerhof et al. 2010). Cryo-electron microscopy analyses have demonstrated the 3D structure of the TAS2R14 and TAS2R16 receptors, thus providing the molecular basis for receptor activation by bitter compounds (Xu et al. 2022; Kim et al. 2024). The bitter off-taste of a limited number of sweeteners has been assessed by cellular-based assays. For example, the activation of TAS2R1 and TAS2R8 has been show to occur with approximately 30 mM cyclamate (Meyerhof et al. 2010). Saccharin and Ace-K activate TAS2R31 and TAS2R43 (Kuhn et al. 2004). A total of 4 TAS2Rs, including TAS2R1, TAS2R10, TAS2R31, and TAS2R46, are activated by sucralose, whereas rebaudioside A (RebA) activates TAS2R4 and TAS2R14 (Hellfritsch et al. 2012; Brune et al. 2013). In addition, it has been shown that sweetener blends reduce bitter off-taste. Cellular assays have demonstrated that cyclamate is capable of inhibiting TAS2R31 and TAS2R43, which are responsible for the bitterness of saccharin, and that saccharin also suppresses the activation of TAS2R1 by cyclamate (Behrens et al. 2017).

One interesting strategy is to identify molecules that are capable of enhancing the perception of sweetness. This compound would not directly induce sweetness but would enhance the sweetness intensity of a small quantity of sweetener or sugar without causing a bitter taste. Only a few synergies have already been examined at the receptor level (Servant et al. 2010; Fujiwara et al. 2012), and it would be relevant to confirm synergies highlighted by sensory analysis to identify new synergies that have never been previously studied. Therefore, this study aimed to investigate the potential activation of sweet and bitter taste receptors by sweeteners alone and binary blends of sweeteners using an in vitro functional assay. To achieve this goal, a total of 9 sweeteners and 6 binary blends were selected. First, 9 sweeteners were tested individually for their activation of TAS1R2/TAS1R3 and were compared to sucrose (used as a control). Second, the ability of these sweeteners to synergize with sweet taste was investigated by using 6 blends. Third, the potential activation of the 25 human TAS2Rs by 9 sweeteners was investigated.

## 2. Materials and methods

### 2.1 The SWEET project

The SWEET project is funded by the European Commission Horizon 2020 (https://sweetproject.eu/). It aims to develop and review evidence on the long-term benefits and potential risks involved in switching over to sweeteners and sweet enhancers in the context of public health and safety, obesity, and sustainability. This 5-year multidisciplinary project involves stakeholders from across the food chain (consumers, patients, health professionals, scientists, policy-makers, and regulators). This study was conducted as part of a work package (WP2—short-term impact on food behavior, physiology & health) that aims to characterize new and emerging sweeteners, identify suitable candidates for the formulation of innovative blends and develop newly reformulated sugar-reduced food products, including individual sweetener and/or sweetener blends.

### 2.2 Sweeteners and blends

A total of 9 sweeteners were selected for this study because of their novelty, safety, correspondence of their sweetness profile with that of sucrose (used as a control) and practicality in certain applications such as beverages or solid foods. Sucrose (natural, ≥ 99.5%), sucralose (E955, synthetic, 99.6%), Ace-K (E950, synthetic, ≥ 99%) and thaumatin (E957, natural, ≥ 99%) were purchased from Sigma-Aldrich (Merck Group; Saint-Quentin-Fallavier, France). RebA (Truvia Stevia Leaf Extract; E960, natural, ≥ 95%), Rebaudioside M (RebM) (Truvia Stevia Leaf Extract; E960, natural, ≥ 80%) and erythritol (Zerose Erythritol; E968, natural, 99.5%) were obtained from Cargill (Cargill R&D Centre Europe BV; Vilvoorde, Belgium). Neotame (E961, synthetic, ≥ 99%) was kindly provided by NutraSweet (Augusta, GA, USA). D-allulose (Allsweet; natural, 99%) and mogroside V (natural, 87.1%) were purchased from Anderson Advanced ingredients (Raalte, Netherlands) and ChromaDex (Longmont, CO, USA), respectively. However, it should be noted that mogroside V and D-allulose both await EFSA approval as of 2024.

Among these 10 sweet compounds, a total of 6 binary blends were selected for the study. These blends are presented in Table 1.

**Table 1.** The examined blends and their concentrations.

Blend no.	Compound 1	Compound 1 concentration	Compound 2	Compound 2 concentration
A	Sucralose	0, 1, 5, 10, 50, 100, 500, 1000 µM	Ace-K	0, 10, 30, 100 µM
B	Neotame	0, 0.05, 0.1, 0.5, 1, 5, 10, 50 µM	D-allulose	0, 3, 10, 30 mM
C	RebA	0, 5, 10, 50, 100, 500, 1000, 5000 µM	Erythritol	0, 3, 10, 30 mM
D	RebM	0, 1, 5, 10, 50, 100, 500, 1000 µM	Mogroside V	0, 0.3, 1, 3, 5, 10 µM
E	Mogroside V	0, 1, 5, 10, 50, 100, 500, 1000 µM	Thaumatin	0, 0.3, 1, 3, 10 µM
F	RebA	0, 5, 10, 50, 100, 500, 1000, 5000 µM	Thaumatin	0, 0.3, 1, 3, 10 µM

RebA, rebaudioside A; RebM, rebaudioside M; Ace-K, acesulfame-K.

### 2.3 Cell-based assays

#### *2.3.1* TAS1R2/TAS1R3 and TAS2R expression constructs.

The sequences encoding TAS1R2, TAS1R3, and the 25 TAS2Rs were commercially synthesized and optimized for expression in mammalian cells. The cDNAs encoding human TAS1R2 and TAS1R3 were subcloned and inserted into the mammalian expression vectors pcDNA6/myc-HisA and pcDNA4/myc-HisA (Invitrogen; Thermo Fisher Scientific; Illkirch, France), respectively, between the *Eco*RI and *Not*I restriction sites, thus generating the pcDNA6-TAS1R2 and pcDNA4-TAS1R3 plasmids. The coding region of the 25 TAS2Rs was individually cloned into the pcDNA4/myc-HisA vector between the *Eco*RI and *Not*I restriction sites, thus generating 25 pcDNA4-TAS2R expression plasmids.

All of the expression plasmids were transformed into the *Escherichia coli* DH5α competent strain by using the heat shock method. The vectors were amplified via bacterial culture in Luria broth media supplemented with ampicillin. Plasmid DNA extraction was performed by using the QIAfilter Plasmid Midi kit (Qiagen; Courtabœuf, France) following the manufacturer’s instructions. The sequences of all the plasmid constructs were verified by using automated DNA sequencing (Genewiz; Leipzig, Germany).

#### 2.3.2 Calcium mobilization assay.

HEK293T cells stably transfected with the chimeric G-protein subunit Gα16gust44 (Ueda et al. 2003; Raliou et al. 2011) were seeded in poly-D-lysine-coated 96-well black plates with clear-bottom plates (0.35 × 10^5^ cells/well) in high-glucose DMEM supplemented with 2 mM GlutaMAX, 10% dialyzed fetal bovine serum, penicillin/streptomycin and G418 (400 μg/ml) at 37°C and 6.3% CO_2_ in a humidified atmosphere. Twenty-four hours later, the cells were transiently transfected with pcDNA6-TAS1R2 and pcDNA4-TAS1R3 (60 ng/well for each plasmid) for sweet taste experiments or with pcDNA4-TAS2R (120 ng/well) for bitter taste experiments, with Lipofectamine 2000 (0.4 μl/well; Invitrogen). As a negative control, HEK293T cells were mock-transfected with the empty expression vector.

After a further 24 h incubation, the HEK293T cells were loaded with the calcium indicator Fluo-4 AM (2.5 μM, Molecular Probes; Invitrogen) dissolved in pluronic acid (0.025%, w/v) in the presence of 2.5 mM probenecid for 1 h at 37 °C. After washing C1 with buffer (130 mM NaCl, 5 mM KCl, 10 mM HEPES pH 7.4, 5 mM sodium pyruvate, 2 mM CaCl_2_), 96-well plates containing the cells were stimulated with sweet-tasting compounds. The fluorescence intensity was measured for 90 s (excitation 488 nm, emission 510 nm) in an automated fluorometric FlexStation3 Multi-Mode microplate reader (Molecular Devices, San Jose, CA, USA).

Different concentration ranges were tested depending on the injected sweet compound. For the blend experiment (Table 1), the 2 sweeteners were mixed together prior to injection. All of the concentration-receptor combinations were measured in triplicate, and each experiment was repeated at least 3 times. The Ca^2+^ change was expressed as a fractional change in fluorescent light intensity: Δ*F*/*F* = (*F* − *F0*)/*F0*, where *F* was the fluorescent light intensity at each point, and *F0* was the value of fluorescent light emitted prior to stimulus application. To calculate dose–response relationships, the changes in fluorescence upon stimulus application were averaged, mock-subtracted and baseline-corrected. The resulting obtained dose–response data were adjusted by using a 4-parameter logistic equation. The half-maximal effective concentration (EC_50_) was calculated by using SigmaPlot software (Systat Software, San Jose, CA, USA) as described in supplemental Supplementary Figs. S1, S2, and S3.

## 3. Results

### 3.1 Activation of sweet taste receptors by sweeteners

**Fig. 1.**
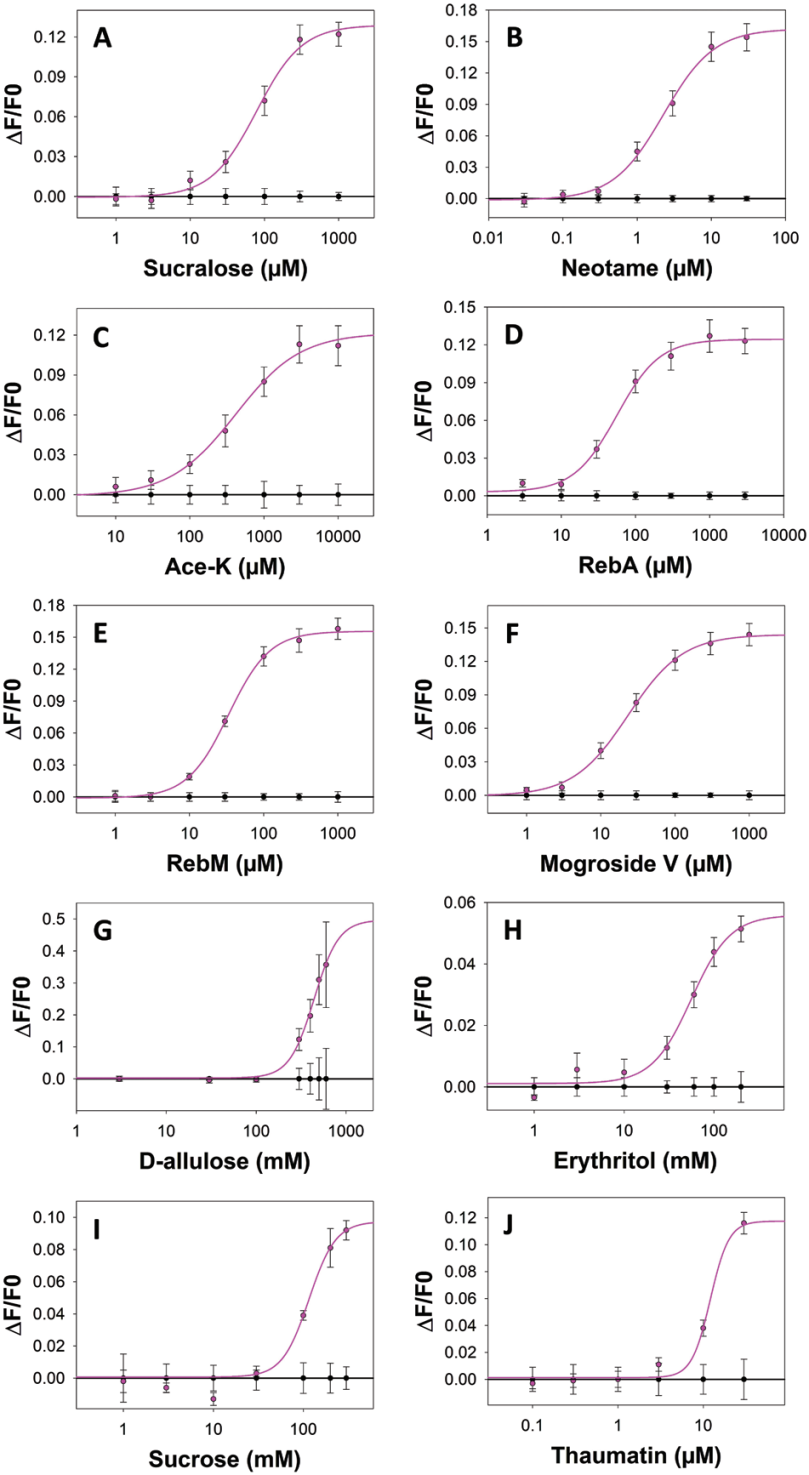
TAS1R2/TAS1R3 dose–response curves measured with sucralose a), neotame b), Ace-K c), RebA d), RebM e), mogroside V f), D-allulose g), erythritol h), sucrose i), and thaumatin j) (pink curves). The control (black curves) corresponds to the dose–response curves of mock-transfected cells (without the receptor). Ace-K: acesulfame-K; RebA: rebaudioside A; RebM: rebaudioside M.

A cell-based assay was performed to determine dose–response relationships for the sweet taste receptor TAS1R2/TAS1R3 by using sucrose and 9 selected sweeteners (Fig. 1). The application of each sweet compound to cells expressing the sweet taste receptor elicited calcium responses in a dose-dependent manner. No response was observed in mock-transfected cells (used as negative controls), except for D-allulose (G), erythritol (H) and sucrose (I) when used at high concentrations. Nonspecific activation was measured for mock-transfected cells but was always lower than in receptor-transfected cells, suggesting that in these cases EC_50_ values could be overestimated and should be taken with caution.

The EC_50_ values were determined for each sweet-tasting compound to compare their effects on functionally expressed TAS1R2/TAS1R3 (Table 2). The lowest EC_50_ value was measured for neotame (2.26 ± 0.23 μM), whereas D-allulose had the highest EC_50_ value (442 ± 78 mM). Sucralose, Ace-K, RebA, RebM, mogroside V and the sweet-tasting protein thaumatin activated the receptor with EC_50_ values in the micromolar range, in contrast to the EC_50_ values for erythritol and sucrose, which were in the millimolar range.

**Table 2.** EC_50_ values, maximal signal amplitude (max Δ*F*/*F0*) and sweetness potency for the different sweeteners.

Sweet compound	EC_50_ value	Max Δ*F*/*F0*	Sweetness potency
Neotame	2.26 ± 0.23 µM	0.16 ± 0.01	10,000^a^
Thaumatin	12 ± 2 µM	0.12 ± 0.01	100,000^b^
Mogroside V	23 ± 2 µM	0.14 ± 0.01	500^c^
RebM	34 ± 2 µM	0.16 ± 0.01	250^d^
RebA	56 ± 6 µM	0.12 ± 0.01	227–439^e,f^
Sucralose	78 ± 11 µM	0.13 ± 0.01	600^g^
Ace-K	410 ± 94 µM	0.12 ± 0.01	200^g^
Erythritol	56 ± 10 mM	0.06 ± 0.01	0.9^f^
Sucrose	116 ± 40 mM	0.10 ± 0.03	1^g^
D-allulose	442 ± 78 mM	0.50 ± 0.12	0.6^h^

^a^ Nofre and Tinti (2000); ^b^van der Wel and Loeve (1972); ^c^Jia and Yang (2009); ^d^Prakash et al. (2014); ^e^Gwak et al. (2012); ^f^Fujimaru et al. (2012); ^g^Schiffman and Gatlin (1993); ^h^Ko et al. (2020).

RebA, rebaudioside A; RebM, rebaudioside M; Ace-K, acesulfame-K.

### 3.2 Activation of the sweet taste receptor by sweetener blends

**Fig. 2.**
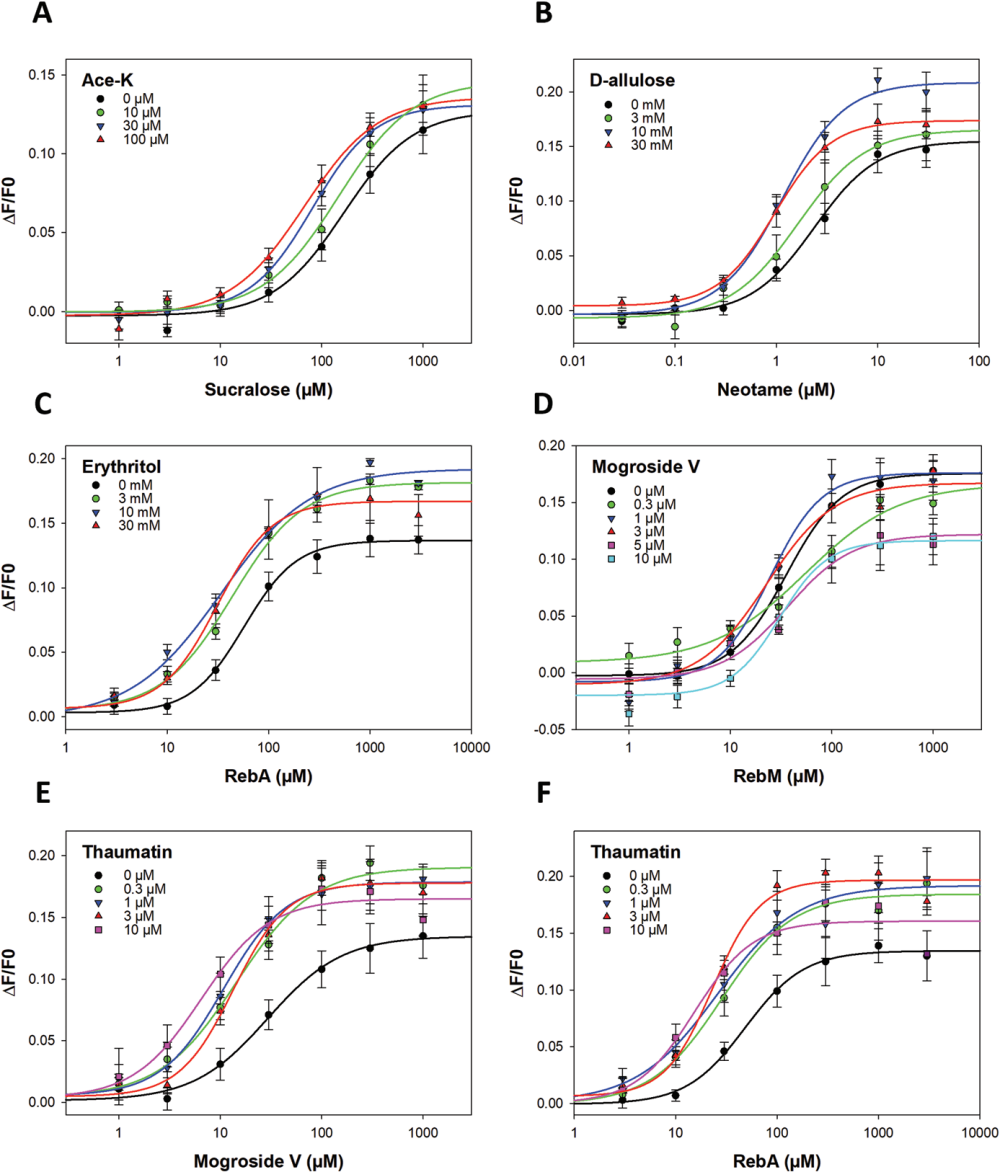
TAS1R2/TAS1R3 dose–response curves were measured with various sweetener blends. The following binary blends were tested: sucralose/Ace-K A), neotame/D-allulose B), RebA/erythritol C), RebM/mogroside V D), mogroside V/thaumatin E), and RebA/thaumatin F). The control (black curve) corresponds to the dose–response curve of the sweetener alone, as indicated on the abscissa. Ace-K: acesulfame-K; RebA: rebaudioside A; RebM: rebaudioside M; Mogroside: mogroside V.

The sweetness intensity of binary blends was investigated by measuring the responses of cultured cells expressing the human sweet taste receptor (TAS1R2/TAS1R3). The concentration-response curves and the EC_50_ values were measured for sucralose, neotame, RebA, RebM, and mogroside V in the presence of different concentrations of another sweetener (Fig. 2; Table 3).

**Table 3.** EC_50_ values and maximal signal amplitude (max Δ*F*/*F0*) for the different binary blends of sweeteners.

Sucralose			Neotame		
Ace-K (µM)	EC_50_ (µM)	Max Δ*F*/*F0*	D-allulose (mM)	EC_50_ (µM)	Max Δ*F*/*F0*
0	165 ± 40	0.13 ± 0.01	0	2.37 ± 0.34	0.16 ± 0.01
10	145 ± 29	0.15 ± 0.01	3	1.63 ± 0.29	0.17 ± 0.01
30	80 ± 10	0.12 ± 0.01	10	1.15 ± 0.13	0.21 ± 0.01
100	67 ± 12	0.14 ± 0.01	30	0.97 ± 0.05	0.17 ± 0.00

RebA			RebM		
Erythritol (mM)	EC_50_ (µM)	Max Δ*F*/*F0*	Mogroside V (µM)	EC_50_ (µM)	Max Δ*F*/*F0*
0	57 ± 5	0.14 ± 0.00	0	36 ± 2	0.17 ± 0.01
3	44 ± 6	0.18 ± 0.01	0.03	55 ± 21	0.16 ± 0.01
10	34 ± 6	0.19 ± 0.01	1	25 ± 5	0.18 ± 0.01
30	31 ± 4	0.17 ± 0.01	3	23 ± 5	0.17 ± 0.01
			5	37 ± 0	0.12 ± 0.01
			10	31 ± 7	0.12 ± 0.01

Mogroside V			RebA		
Thaumatin (µM)	EC_50_ (µM)	Max Δ*F*/*F0*	Thaumatin (µM)	EC_50_ (µM)	Max Δ*F*/*F0*
0	29 ± 4	0.14 ± 0.01	0	49 ± 5	0.13 ± 0.00
0.03	14 ± 3	0.19 ± 0.01	0.3	28 ± 4	0.18 ± 0.01
1	11 ± 1	0.18 ± 0.04	1	26 ± 7	0.19 ± 0.01
3	13 ± 2	0.18 ± 0.01	3	23 ± 4	0.20 ± 0.01
10	7 ± 2	0.17 ± 0.01	10	15 ± 5	0.16 ± 0.01

Ace-K, acesulfame-K; RebA, rebaudioside A; RebM, rebaudioside M.

The sucralose-mediated stimulation of TAS1R2/TAS1R3 receptor in the presence of increasing concentrations of Ace-K (0 to 100 µM) resulted in a leftward shift of the dose–response curve and a decrease in the EC_50_ values (Fig. 2a; Table 3). In a similar experiment, the dose–response curves of neotame in the presence of increasing concentrations of D-allulose (0 to 30 mM) were measured (Fig. 2b; Table 3). The addition of D-allulose resulted in a left shift in the dose–response curves of neotame. The maximal signal amplitude was slightly increased for 10 mM D-allulose. The dose–responses curve for RebA in the presence of different concentrations of erythritol (0 to 30 mM) showed a decrease of 54% to 77% in the EC_50_ values and an increase of approximately 21% to 35% in the maximal amplitude of the response (Fig. 2c; Table 3). The EC_50_ values of RebM were similar for each concentration of mogroside V (0 to 30 µM) (Fig. 2d; Table 3). For 5 and 10 µM mogroside V, the maximal amplitude of the response decreased. The addition of the sweet-tasting protein thaumatin (0 to 10 µM) significantly decreased the EC_50_ values of mogroside V (Fig. 2e; Table 3). For example, a 4-fold decrease in the EC_50_ value of mogroside V was observed in the presence of 10 µM thaumatin. In addition, for all of the tested concentrations of thaumatin, the maximal signal amplitude also increased from 21% to 35%. Finally, the dose–response curves of RebA in the presence of increasing concentrations of thaumatin (0 to 10 µM) were measured (Fig. 2f; Table 3). A leftward shift of the dose–response curves and a 2-fold decrease in EC_50_ values were observed when 0.3, 3 or 1 µM thaumatin was added to RebA. Moreover, the maximal signal amplitude was also increased by more than 38%.

### 3.3 Activation of bitter taste receptors by sweeteners and blends

The activation of the 25 human TAS2Rs by the 9 selected sweeteners (excluding sucrose) was examined by using HEK293T-Gα16gust44 cells. Cellular responses were measured after cell stimulation with various concentrations of the sweeteners. The response profiles of the 25 TAS2Rs stimulated with the examined compounds are presented in Table 4. Neither neotame, RebM, erythritol, or thaumatin activated any TAS2Rs at the maximal tested concentration, whereas the other sweeteners activated a limited number of bitter taste receptors.

**Table 4.** Response profiles of the 25 TAS2Rs stimulated with the selected sweeteners.

Receptors	Studied sweeteners (maximal concentration tested)								
	Sucralose 100 mM	Neotame 30 µM	Ace-K 300 mM	RebA 4 mM	RebM 4 mM	Mogroside V 5 mM	Erythritol 200 mM	D-allulose 500 mM	Thaumatin 100 mM
TAS2R1	#^a^	−	−	−	−	−	−	−	−
TAS2R3	−	−	−	−	−	−	−	−	−
TAS2R4	−	−	−	#^c^	−	−	−	−	−
TAS2R5	−	−	−	−	−	−	−	−	−
TAS2R7	−	−	−	−	−	−	−	−	−
TAS2R8	−	−	−	−	−	−	−	−	−
TAS2R9	−	−	−	−	−	−	−	−	−
TAS2R10	#^a^	−	−	−	−	−	−	−	−
TAS2R13	−	−	−	−	−	−	−	−	−
TAS2R14	−	−	−	#^c^	−	−	−	**+**	−
TAS2R16	−	−	−	−	−	**+**	−	**+**	−
TAS2R19	−	−	−	−	−	−	−	**+**	−
TAS2R20	−	−	−	−	−	−	−	−	−
TAS2R30	−	−	−	−	−	−	−	**+**	−
TAS2R31	§^a^	−	#^b^	−	−	−	−	−	−
TAS2R38	−	−	−	−	−	**+**	−	**+**	−
TAS2R39	−	−	−	−	−	−	−	−	−
TAS2R40	−	−	−	−	−	−	−	−	−
TAS2R41	−	−	−	−	−	−	−	−	−
TAS2R42	−	−	−	−	−	−	−	**+**	−
TAS2R43	−	−	§^b^	−	−	−	−	**+**	−
TAS2R45	−	−	−	−	−	−	−	−	−
TAS2R46	#^a^	−	−	−	−	−	−	−	−
TAS2R50	−	−	−	−	−	−	−	−	−
TAS2R60	−	−	−	−	−	−	−	−	−

^a^ Brune et al. (2013); ^b^Kuhn et al. (2004); ^c^Hellfritsch et al. (2012).

“+”: response; “−”: no response; “#”: previously known agonist confirmed in the present study; “§”: previously known agonist not confirmed in the present study. Abbreviations: Ace-K, acesulfame-K; RebA, rebaudioside A; RebM, rebaudioside M.

**Fig. 3.**
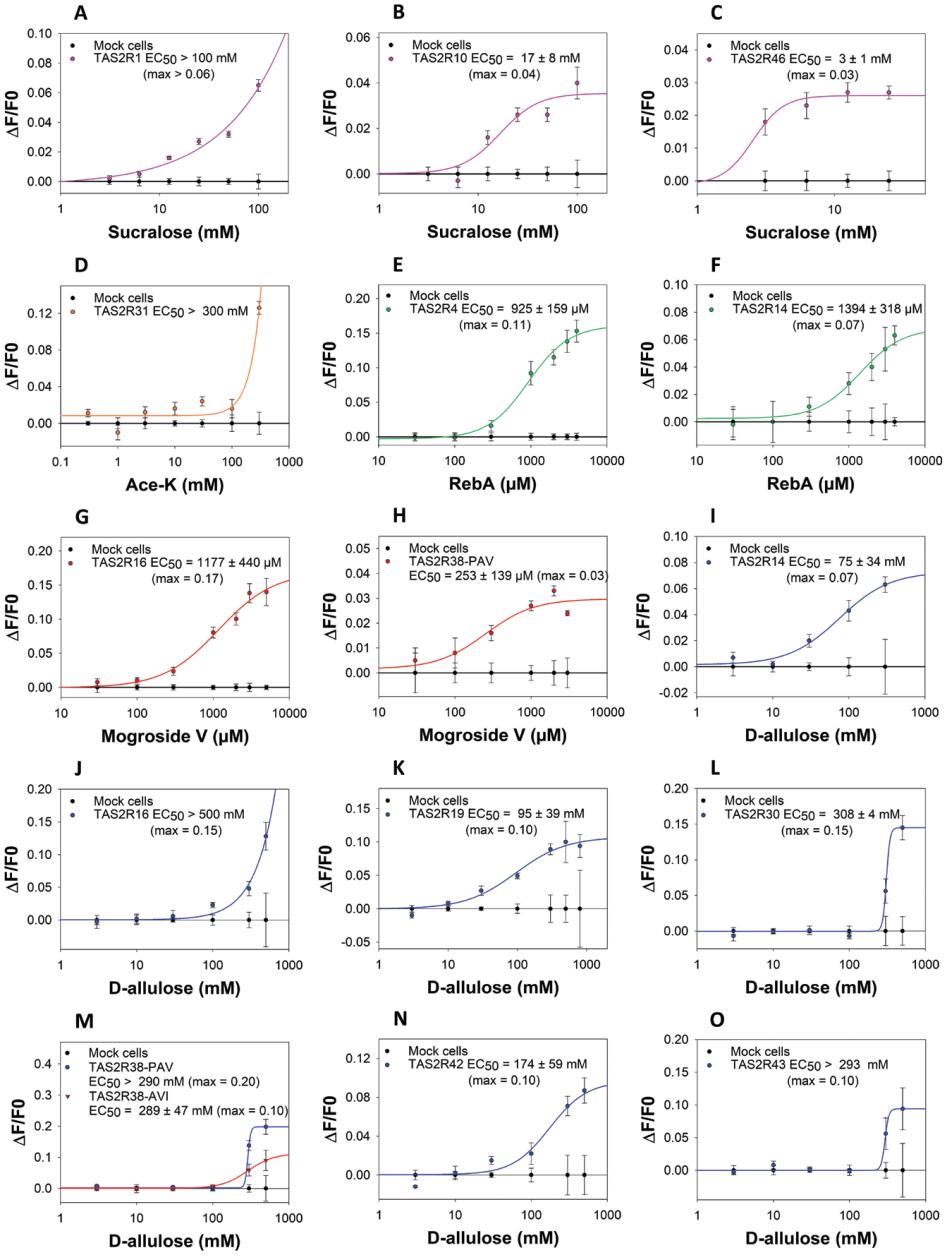
Dose–response curves for TAS2R1, TAS2R10, and TAS2R46 for sucralose (pink curves) A–C), TAS2R31 for Ace-K (orange curve) D), TAS2R4 and TAS2R14 for Reb A (green curves) E and F), TAS2R10 and TAS2R38-PAV for mogroside V (red curves) G and H) and TAS2R14, TAS2R16, TAS2R19, TAS2R30, TAS2R38-PAV/AVI, TAS2R42, and TAS2R43 for D-allulose (blue and red curves) I–O). The control (black curves) corresponds to the dose-response curves of mock-transfected cells (without the receptor). Abbreviations: Ace-K, acesulfame-K; RebA, rebaudioside A.

Subsequently, we measured the dose–response curves and determined the EC_50_ values for TAS2Rs activated by sucralose, Ace-K, RebA, mogroside V, and D-allulose (Fig. 3).

Sucralose activated 3 TAS2Rs with different EC_50_ values, including TAS2R46 (EC_50_ = 3 ± 1 mM) and TAS2R10 (EC_50_ = 17 ± 8 mM) with similar EC_50_ values, whereas for TAS2R1, the tested concentrations were not high enough to fully activate the receptor and determine its EC_50_ value. However, this concentration could be estimated to be greater than 100 mM (Fig. 3a–c). In contrast with a previous study (Brune et al. 2013), we did not observe activation of TAS2R31, even after several trials. Contrary to previous results (Kuhn et al. 2004), Ace-K activated TAS2R31 (EC_50_ > 300 mM, Fig. 3d) but not TAS2R43. RebA activated the TAS2R4 and TAS2R14 receptors (Fig. 3e and f). RebA elicited TAS2R4 (EC_50_ = 925 ± 159 µM) and TAS2R14 (EC_50_ = 1394 ± 318 µM) responses, as previously reported (Hellfritsch et al. 2012), for concentrations above 100 µM which were close to those corresponding to the saturation of the sweet taste receptor signal. Mogroside V activated 2 TAS2Rs with different EC_50_ values of 253 ± 139 and 1177 ± 440 µM for TAS2R38 and TAS2R16, respectively (Fig. 3g and h). Surprisingly, a total of 7 TAS2Rs were activated by D-allulose (Fig. 3i–o). TAS2R14, TAS2R19, and TAS2R42 were the most sensitive receptors, with EC_50_ values less than 200 mM, whereas TAS2R16, TAS2R30, TAS2R43, TAS2R38-PAV, and TAS2R38-AVI were activated at D-allulose concentrations greater than 300 mM. No difference was found in the EC_50_ values for TAS2R38-PAV and TAS2R38-AVI, but the maximal amplitude of the signal was reduced for TAS2R38-AVI. It would be interesting to confirm this observation with a sensory study and determine whether there is a difference in D-allulose detection threshold between individuals carrying PAV/PAV and AVI/AVI haplotypes (Hayes et al. 2008; Behrens et al. 2013). Indeed, a few rare studies have demonstrated the functional activation of the TAS2R38-AVI receptor (Karolkowski et al. 2023). Nevertheless, due to low nonspecific activation of mock-transfected cells by D-allulose at high concentrations, the extrapolated EC_50_ values calculated for the activated TAS2Rs should be taken with caution.

## 4. Discussion

First, we tested the ability of sucrose and 9 selected sweeteners alone to activate the sweet taste receptor. The dose–responses of TAS1R2/TAS1R3 and the measured EC_50_ values were similar to those that have been previously published (Xu et al. 2004; Servant et al. 2010; Ohta et al. 2011; Belloir et al. 2021; Choi et al. 2021), except for those of erythritol and mogroside V, for which no data were available in the literature. Interestingly, the EC_50_ value of RebA (EC_50_ = 56 ± 6 µM) was greater than that of RebM (EC_50_ = 34 ± 2 µM), whereas the sweetness of RebA may be similar to or greater than that of RebM (Table 2) (Prakash et al. 2014). One hypothesis to explain this discrepancy could be the use of different extraction methods for these steviosides, thus leading to differences in quality or purity. Moreover, the presence of 2 additional sugars in RebM (Prakash et al. 2014) could explain its greater affinity for the TAS1R2-VTF domain compared with RebA (Zhang et al. 2010). We found that the EC_50_ values of all of the tested molecules were correlated with their sweetness potency (Table 2), as has been previously described (Choi et al. 2021); thus, a lower EC_50_ value indicated a greater sweetness potency. These cellular data confirmed that the following sweeteners (ordered by increasing sweetness potency), including neotame, thaumatin, mogroside V, RebM, RebA, sucralose, Ace-K, and erythritol, could be used alone to replace sucrose while reducing caloric intake. However, the type of food application must be considered, as sucrose and D-allulose also have a texturizing role in the matrix (Erickson and Carr 2020).

**Fig. 4.**
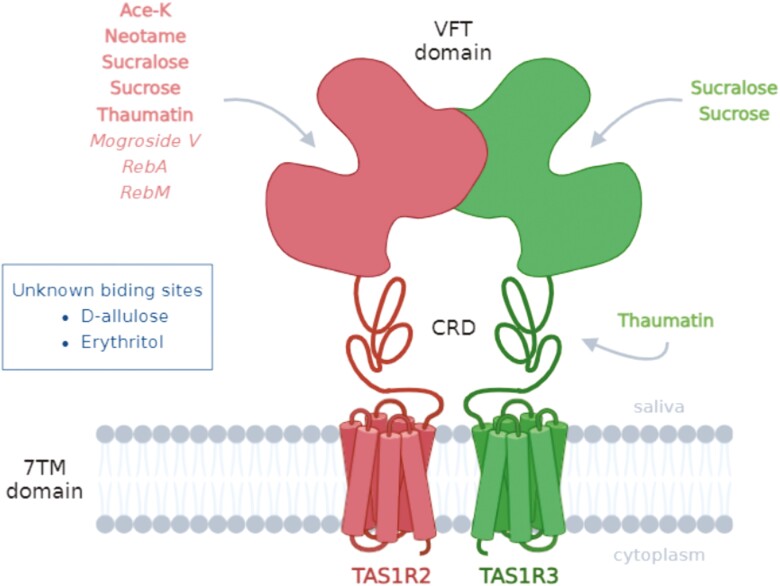
Schematic summary of the different sweet taste receptor (TAS1R2/TAS1R3) binding sites of the examined sweeteners. The sweeteners in which binding sites are known are in bold (Jiang et al. 2004; Xu et al. 2004; Nie et al. 2005; Maîtrepierre et al. 2012; Masuda et al. 2013), whereas the binding sites of the sweeteners in italics are hypothetical (Zhang et al. 2010; Kim et al. 2017). Abbreviations: Ace-K, acesulfame-K; VFT, Venus flytrap domain; CRD, cysteine-rich domain; 7TM, 7-helix transmembrane.

Second, the ability of the 6 binary blends to activate the sweet taste receptor was tested. The decrease in the EC_50_ values of sucralose upon the addition of increasing concentrations of Ace-K (Table 3) demonstrated a greater sweetener potency for the sucralose and Ace-K blend than for sucralose alone. The data confirmed that sucralose and Ace-K were synergistic at the receptor level, which was consistent with the results of previous sensory studies (Schiffman et al. 1995a; Reyes et al. 2019). The multiple binding sites on the sweet taste receptor for sucralose and Ace-K likely explain the observed synergy. Sucralose and Ace-K interact with the VFT domain of TAS1R2, whereas the TAS1R3-VFT domain also binds to sucralose (Xu et al. 2004; Nie et al. 2005; Maîtrepierre et al. 2012). The addition of D-allulose (3 to 30 mM), which did not activate the sweet taste receptor at these concentrations (Fig. 1), resulted in a leftward shift of the dose–response curves of neotame, thus corresponding to an approximately 2.5-fold increase in sweetness potency (Fig. 2b; Table 3). In addition, the maximal signal amplitude, which measures the efficacy of the response, was slightly increased for 10 mM D-allulose. These results confirmed the synergistic effect between neotame and D-allulose. Although neotame binds to TAS1R2-VFT (Xu et al. 2004), the binding sites of the TAS1R2/TAS1R3 receptor are not known for D-allulose. This synergism suggested that D-allulose bound to a different site than TAS1R2-VFT. The dose–response curve for RebA in the presence of different concentrations of erythritol showed a 1.3 to 1.8-fold increase in sweetness potency and an increase in efficacity of approximately 20% (Table 3). The synergistic effect on sweetness potency was less than that observed for sucralose/Ace-K and neotame/D-allulose but was more effective with 3 mM erythritol, with a 22% decrease in EC_50_ values. This synergistic effect suggested that RebA and erythritol should interact with at least one different TAS1R2/TAS1R3 binding site. Moreover, this result was consistent with a previous sensory study that showed a synergistic effect. For example, a concentration of 0.018 g of RebA and 48 g of erythritol per kg of water provided a sweetness that was 38 times greater than that of a 3% sucrose solution (Heikel et al. 2012). The addition of mogroside V did not increase the sweetness potency or the efficacy of RebM (Fig. 2d), thus suggesting that no synergy was observed for this sweetener blend. Prediction of the 3D structure of TAS1R2/TAS1R3 suggested that mogroside V could bind to TAS1R2-VFT (Kim et al. 2017). In addition, steviosides were able to bind to TAS1R2-VFT (Zhang et al. 2010), thus suggesting that Reb M bonded to the same sweet taste receptor activation site as mogroside V. However, this hypothesis remains to be validated by combining cellular assays and site-directed mutagenesis. Conversely, the addition of thaumatin significantly increased the sweetness potency and the efficacy of mogroside V (Fig. 2e; Table 3) and RebA (Fig. 2f; Table 3). For concentrations of thaumatin ranging from 0.3 to 10 µM, a 3-fold and 2-fold decrease in the EC_50_ values of mogroside V and RebA, respectively, were detected in the leftward shift of the dose–response curves. The efficacy of mogroside V also increased from 21% to 35% and by more than 38% for RebA when blended with thaumatin, thus demonstrating a synergistic effect between these sweeteners. Interestingly, these synergistic effects occurred with thaumatin at concentrations less than 3 µM, which was unable to activate the sweet taste receptor on its own at this concentration (Fig. 1). Surprisingly, this result was not consistent with a previous sensory study that showed that a blend of thaumatin (25% to 75%) and RebA (25% to 75%) had a suppressive effect on the sweet intensity (Reyes et al. 2019). However, in a blend of 99.00% RebA and 0.01% thaumatin a synergistic effect was observed via sensory analysis (Schiffman et al. 2003). This difference could be explained by the excessive concentration of thaumatin in the blend. Indeed, our data showed that the synergistic effect was less pronounced for 10 µM thaumatin and that concentrations of 0 to 3 µM thaumatin corresponded to 0% to 11% thaumatin in the RebA/thaumatin blend. Finally, these 2 synergistic effects could be explained by the activation of a different site of the sweet taste receptor by mogroside V or RebA and thaumatin. Indeed, RebA, which is a stevioside, and mogroside V have been shown to bind TAS1R2-VTF (Zhang et al. 2010; Kim et al. 2017), whereas thaumatin interacts with TAS1R3-CRD (Masuda et al. 2013). Finally, the combination of 2 sweeteners was particularly useful for improving the sweetness potency of a single compound while limiting its initial concentration. Our cellular data confirmed the synergistic effects of 3 blends that have been previously identified in the literature (sucralose/Ace-K, RebA/erythritol, and RebA/thaumatin) and identified 2 new blends, including neotame/D-allulose and mogroside V/thaumatin. However, the RebM/mogroside V blend did not have a synergistic effect on sweetening. The synergy of a blends was observed when the compounds shared at least one different binding site, although this hypothesis had to be validated by determining the binding site(s) of the following molecules: erythritol, mogroside V, and D-allulose. A summary of the taste receptor binding sites of the tested sweeteners is shown in Fig. 4. A better understanding of the binding sites and the molecular mechanisms of activation of the sweet taste receptor can provide an avenue for studying the synergistic effect of even more elaborate molecules, such as blends combining 3 or even 4 sweeteners, which have already been investigated in sensory analysis (Schiffman et al. 2000, 2003, 2007; Jang et al. 2021). However, the addition of sweeteners could also lead to the perception of off-flavors, such as bitterness.

Third, the ability of 9 sweeteners alone to activate the 25 human TAS2Rs was tested. Our data confirmed the activation of bitter taste receptors by some sweeteners, including Ace-K, RebA, and sucralose, and highlighted for the first time the TAS2R activation profile of mogroside V and D-allulose. Moreover, these results were consistent with previous sensory analyses of these sweeteners (Schiffman et al. 1995b; Fujimaru et al. 2012; Allen, McGeary, Knopik et al. 2013; Laffitte et al. 2016; Tan et al. 2019). The activation of TAS2R1, TAS2R10, and TAS243 by sucralose was consistent with the finding of a previous study, except for TAS2R31, which had already been observed to be activated by sucralose (Brune et al. 2013). Interestingly, a small quantity of sucralose in a food product should be sufficient to activate TAS1R2/TAS1R3 (EC_50_ = 78 ± 11 µM, maximal signal amplitude reached below 1 mM; Fig. 1a; Table 2) without activating the TAS2R1, TASR10 and TAS1R46 receptors (EC_50_ ≥ 3 mM; Fig. 3a–c). This observation was consistent with an earlier in vivo study showing the perception of sucralose bitterness only at high concentrations (12.6 and 50.18 mM) (Antenucci and Hayes 2015). Ace-K activated TAS2R31 (EC_50_ > 300 mM, Fig. 3d) but not TAS2R43, as has been demonstrated in a previous study (Kuhn et al. 2004). The lack of activation of TAS2R43 could be explained by differences in the receptor sequence (polymorphism). However, the concentration at which Ace-K activated TAS2R31 was much greater than that at which it stimulated the sweet taste receptor (EC_50_ = 410 ± 94 µM). Thus, although both sucralose and Ace-K activated TAS2R31, the addition of 100 µM Ace-K to sucralose improved the sweetness potency of sucralose while preventing the activation of TAS2Rs (Fig. 2a). RebA activated TAS2R4 and TAS2R14 (Fig. 3e and f), as has been previously reported using cell-based assays, human sensory analysis and molecular docking (Hellfritsch et al. 2012; Acevedo et al. 2016; Singla and Jaitak 2016). Based on a previous structure-function study that was performed on isoflavones and TAS2Rs, the lower number of glucosides in RebA than in RebM could explain why the latter type did not activate TAS2R14. Indeed, it has already been shown that the glycosylation of isoflavones inhibits the activation of this bitter receptor (Roland et al. 2011). A blend of RebA and erythritol or thaumatin, which did not activate TAS2Rs, reduced the EC_50_ value of RebA for TAS1R2/TAS1R3 (Fig. 3c and e) while preventing the activation of bitter receptors. The sweetener mogroside V activated TAS2R16 and TAS2R38. The presence of a few β-glucopyranoside moieties should explain the recognition of mogroside V by TAS2R16 (Bufe et al. 2002). The EC_50_ values for TAS2R16 (EC_50_ = 1177 ± 440 µM) and TAS2R38 (EC_50_ = 253 ± 139 µM) were greater than that of TAS1R2/TAS1R3 (EC_50_ = 23 ± 2 µM), but the saturation of the sweet taste receptor reached approximatively 500 µM (Fig. 1), which could explain the perception of sweet and bitter tastes of mogroside V used at high concentrations in the blend formulation. Moreover, the addition of thaumatin increased the sweetness potency of mogroside V (Fig. 2e), thus suggesting that a smaller quantity of mogroside V (less than 100 µM) in a blend contributed to an interesting sweetness potency while avoiding the activation of TAS2Rs. In addition, there are several polymorphisms for these 2 bitter receptors activated by mogroside V (Bufe et al. 2005; Soranzo et al. 2005), therefore, it would be relevant to validate whether there are sensory differences in the bitter taste perception of mogroside V among different populations. We found that D-allulose activated 7 TAS2Rs. As the EC_50_ of D-allulose for the sweet taste receptor was 442 mM (Table 2) and below 200 mM for 3 TAS2Rs (Fig. 3), these results suggested that D-allulose used at high concentrations could induce both bitterness and a sweet taste. A 13.31% D-allulose solution (approximately 740 mM) has previously been described as being bitter in sensory analysis (Tan et al. 2019), which was consistent with the EC_50_ values that were obtained in our study. However, the use of 10 mM D-allulose in a blend with neotame increased the sweetness potency of this latter compound (Fig. 2b) without activating any TAS2Rs. It should be noted that D-allulose activated bitter taste receptors common to RebA (TAS2R14), mogroside V (TAS2R16 and TAS2R38) and Ace-K (TAS2R43), therefore, these blends should not be selected because they are likely be associated with bitter taste. However, this remains to be verified and depends on the concentrations that are tested. Finally, the concentration of these sweeteners should be precisely determined to limit the activation of bitter taste receptors while ensuring the activation of sweet taste receptor. The choice of blends of these sweeteners should also be appropriate for limiting the presence of bitterness in food products or beverages.

## 5. Conclusion

Our data provide a receptor-based understanding of the complex synergies of sweet taste and an effective approach for testing sweeteners either alone or in combination while avoiding the activation of TAS2Rs, which could reduce the sugar content in foods. This method will be useful for designing new sweetener blends with reduced off-taste.

Synergy was observed for 5 of the 6 tested blends. These synergistic effects could be explained by the activation of at least one binding site on the sweet taste receptor by the 2 molecules in the blend. Based on these data, the following blends appeared to be the most interesting for increasing the sweetness intensity while limiting the bitterness perception and caloric intake of food products: RebA/erythritol, mogroside V/thaumatin, and RebA/thaumatin. The combination of 2 sweeteners made it possible to increase the sweetness potency of the compound alone due to a synergistic effect and also to limit the bitter taste of the blend, due to the fact that the EC_50_ values of TAS1R2/TAS1R3 were most often observed below the activation threshold of TAS2Rs. However, the selection of these 3 blends and concentration adjustments should be confirmed by a human sensory study. Finally, other parameters must also be considered when choosing a blend, including the texturizing properties of these sugars, the presence of off-flavors other than bitterness (liquorice, menthol, metallic, and astringent flavors, among others), the EFSA approval and their potential harm (Kroger et al. 2006; Laffitte et al. 2016; Erickson and Carr 2020).

## Supplementary Material

bjae041_suppl_Supplementary_Materials

## Data Availability

The data underlying this article will be shared on reasonable request to the corresponding author.
