# Supplementary material for: A receptor-based assay to study the sweet and bitter tastes of sweeteners and binary sweet blends: the SWEET project
Source: Chem Senses. 2024 Nov 8;49:bjae041. doi: 10.1093/chemse/bjae041 (PMC11631053; doi:10.1093/chemse/bjae041)
Supplement: bjae041_suppl_Supplementary_Materials [file bjae041_suppl_supplementary_materials.docx]

**A receptor-based assay to study the sweet and bitter tastes of sweeteners and binary sweet blends: The SWEET Project – Supplementary data**

Christine Belloir, Mathilde Jeannin, Adeline Karolkowski, Corey Scott, Loïc Briand

^
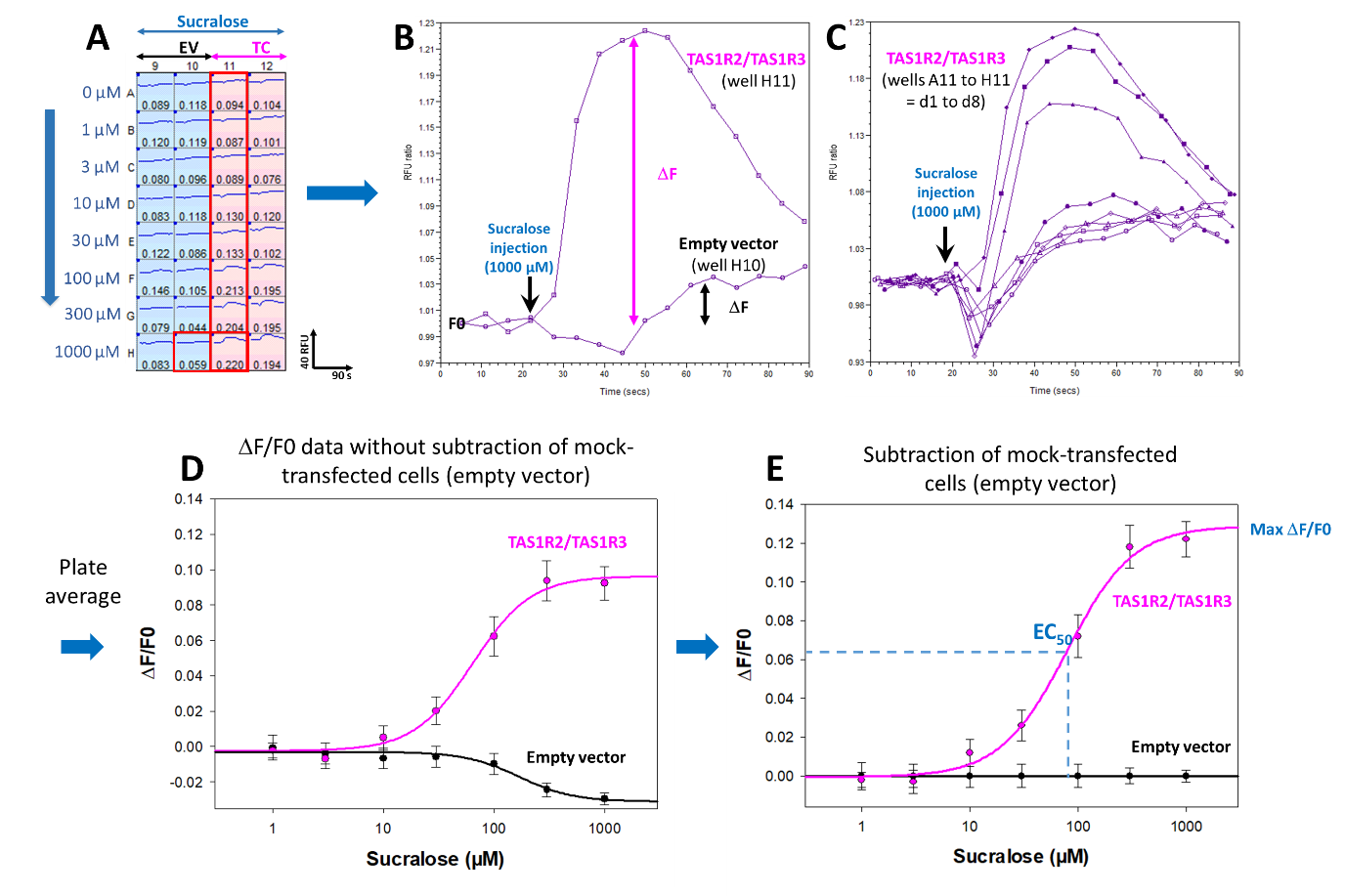
^

**Figure S1.** Example of data analysis of calcium kinetics. **(A)** HEK 293T-Gα16gust44 cells transfected with human TAS1R2/TAS1R3 (pink wells) or empty vector controls (blue wells) responded to stimulation with increasing concentrations of sucralose (0-1 000 µM). Blue traces show the fluorescence changes of cells stimulated, value indicates the ratio ΔF/F0 with ΔF the variation of fluorescence at maximal amplitude and F0 the mean of the fluorescence during the first 20 s. Scale bars are shown at the bottom. **(B)** Raw fluorescence traces of cells transfected with TAS1R2/TAS1R3 (purple squares) and empty vector controls (purple circles) stimulated with 1 000 µM sucralose (wells H11 and H10, respectively). **(C)** Raw fluorescence traces of cells transfected with TAS1R2/TAS1R3 stimulated by a range of sucralose (0 µM to 1 000 µM, wells A11 to H11). Stimulus application is indicated by a black arrow. **(D)** Changes in fluorescence upon stimulus application were averaged with plate replicate and baseline corrected to plot the graphs using SigmaPlot software. **(E)** Finally, mock-transfected cells (empty vector) were subtracted and dose-response data were adjusted by using four-parameter logistic (4PL) equation to calculate EC_50_ concentrations and maximal ΔF/F0 using SigmaPlot software. EV: empty vector; EC_50_: half-maximal effective concentration.


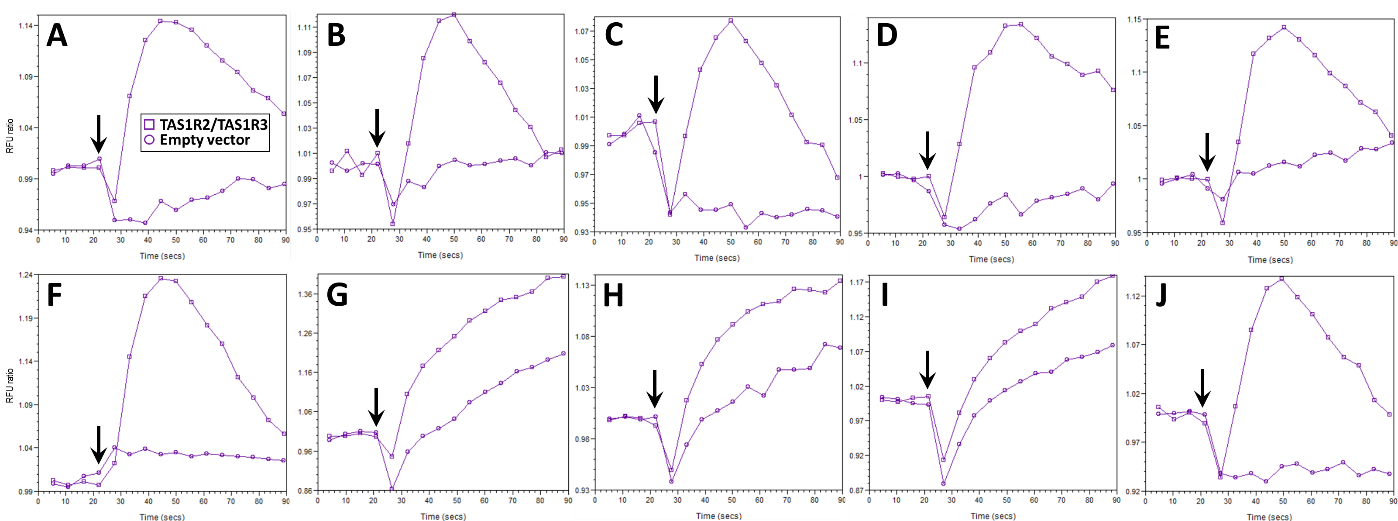


**Figure S2.** Example of calcium traces recorded in the FlexStation after stimulation of the cells with 10 sweet tasting compounds. Raw fluorescence traces of HEK 293T-Gα16gust44 cells transfected with TAS1R2/TAS1R3 plasmid (purple squares) or empty vector (purple circles) stimulated with **(A)** 1 000 µM sucralose, **(B)** 30 µM neotame, **(C)** 10 mM Ace-K, **(D)** 3 000 µM RebA, **(E)** 1 000 µM RebM, **(F)** 1 000 µM mogroside V, **(G)** 400 mM D-allulose, **(H)** 300 mM erythritol, **(I)** 300 mM sucrose and **(J)** 30 µM thaumatin. Stimulus application is indicated by a black arrow. Scale: y-axis = relative fluorescence units (RFU) ratio; x-axis = time (secs). Ace-K: acesulfame-K; RebA: rebaudioside A; RebM: rebaudioside B.


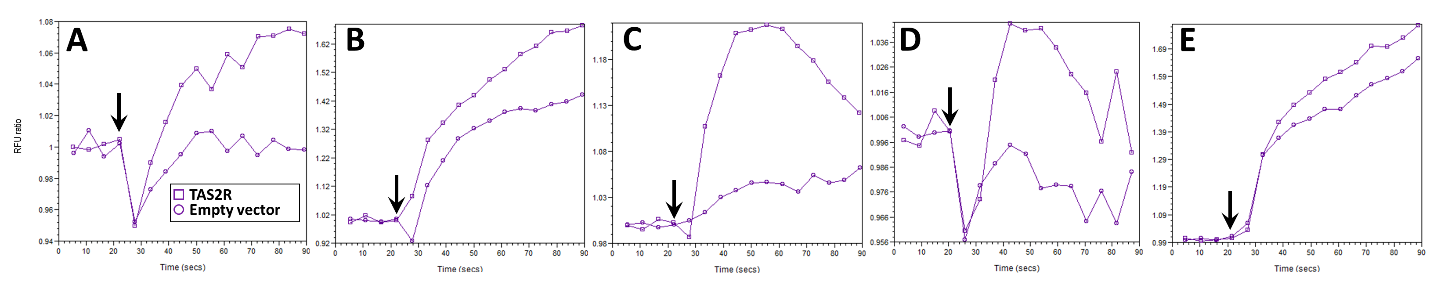


**Figure S3.** Example of calcium traces recorded in the FlexStation after stimulation of the cells with bitter compounds. Raw fluorescence traces of HEK 293T-Gα16gust44 cells transfected with TAS2R plasmid (purple squares) or empty vector (purple circles) stimulated with: **(A)** TAS2R1 + 100 mM sucralose, **(B)** TAS2R31 + 300 mM Ace-K, **(C)** TAS2R4 + 4 mM RebA, **(D)** TAS2R38 and 2 000 µM mogroside V and **(E)** TAS2R14 + 300 mM D-allulose. Stimulus application is indicated by a black arrow. Scale: y-axis = relative fluorescence units (RFU) ratio; x-axis = time (secs). Ace-K: acesulfame-K; RebA: rebaudioside A.
